# Supplementary material for: Advancing Nanoscale Science: Synthesis and Bioprinting of Zeolitic Imidazole Framework-8 for Enhanced Anti-Infectious Therapeutic Efficacies
Source: Biomedicines. 2023 Oct 18;11(10):2832. doi: 10.3390/biomedicines11102832 (PMC10604899; doi:10.3390/biomedicines11102832)

SI data

Advancing Nanoscale Science: Synthesis and Bioprinting of Zeolitic Imidazole Framework-8 for Enhanced Anti-infectious Therapeutic Efficacies

Muhammad Saqib Saif[1], Murtaza Hasan[2,3*], Ayesha Zafar[4], Muhammad Mahmood Ahmad[5], TubaTariq[1], Muhammad Waqas[2], Riaz Hussain[6], Amina Zafar[5], Xue Huang[3], Xugang Shu[3***]**

[1]Faculty of Chemical and Biological Science, Department of Biochemistry, The Islamia University of Bahawalpur, 63100, Pakistan

[2]Faculty of Chemical and Biological Science, Department of Bioctechnlogy, The Islamia University of Bahawalpur, 63100, Pakistan

[3]School of Chemistry and Chemical Engineering, Zhongkai University of Agriculture and Engineering, Guangzhou, 510225, P.R. China

[4]School of Engineering, Royal Melbourne Institute of Technology (RMIT) University Melbourne, 124 La Trobe Street, Melbourne, VIC 3001, Australia

[5]Faculty of Chemical and Biological Science, Department of Bioinformatics, The Islamia University of Bahawalpur, 63100, Pakistan

[6]Faculty of Chemical and Biological Science, Department of Veterinary Sciences, The Islamia University of Bahawalpur, 63100, Pakistan

AUTHOR INFORMATION

Corresponding Authors:

Dr Murtaza Hasan[*]

Email: murtaza@zhku.edu.cn

Prof Xugang Shu[**]

Email: xgshu@21cn.com

Phone: 86-020-8900-3114.

Fax: 86-020-8900-3114

Table S1. (a,b) Nano-size calculation using Scherrer equation (a) ZIF-8, (b) CME@ZIF-8.

(a)

Peak number	Pos. (2θ)	Intensity	FWHM left (2θ)	β (rad)	Size (nm)	Average size (nm)
1	7.8086	118.336	10.07412	0.175826	0.789308	11.38424
2	10.24216	724.617	0.45702	0.007976	17.45247	
3	12.74844	717.996	0.35112	0.006128	22.76631	
4	14.71232	1247.707	0.59133	0.010320	13.5461	
5	18.64383	711.375	0.53906	0.009408	14.93453	
6	20.07974	114.431	0.65821	0.011487	12.24421	
7	20.07974	61.460	61.21047	1.068324	0.311805	
8	27.14889	485.738	0.62562	0.010919	13.06318	
9	29.77154	223.938	1.09162	0.019052	7.530238	

(b)

Peak number	Pos. (2θ)	Intensity	FWHM left (2θ)	β (rad)	Size (nm)	Average size (nm)
1	10.1201	701.612	0.38386	0.006699	20.22677	12.4482
2	12.99014	719.650	0.5258	0.009176	15.20656	
3	14.33409	2659.380	0.36962	0.006451	21.66238	
4	18.21586	723.896	0.38988	0.006804	20.63643	
5	18.21586	48.599	8.8834	0.155044	0.905704	
6	18.21586	145.161	1134.3144	19.797521	0.007093	
7	26.78703	289.387	0.42156	0.007357	19.37185	
8	30.00994	26.315	8.07282	0.140897	1.018817	

Figure S1. EDX analysis of **(a)**ZIF-8, **(b)** CME@ZIF-8

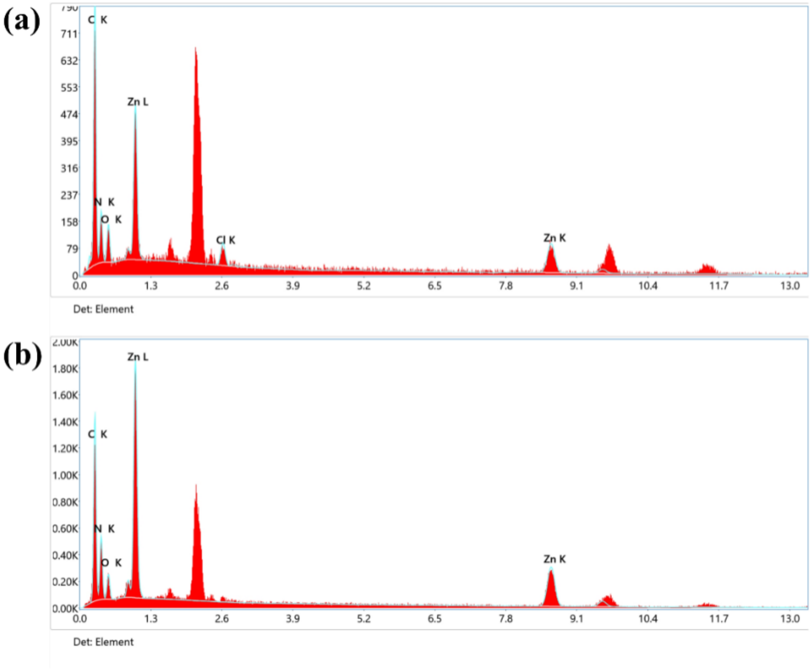

Supplement: Supplementary file 1 [file biomedicines-11-02832-s001.zip › biomedicines-2625863-supplementary.pdf]
